# Identification of novel candidate target genes in amplicons of Glioblastoma multiforme tumors detected by expression and CGH microarray profiling

**DOI:** 10.1186/1476-4598-5-39

**Published:** 2006-09-26

**Authors:** Yolanda Ruano, Manuela Mollejo, Teresa Ribalta, Concepción Fiaño, Francisca I Camacho, Elena Gómez, Angel Rodríguez de Lope, Jose-Luis Hernández-Moneo, Pedro Martínez, Bárbara Meléndez

**Affiliations:** 1 Genetics Department, Hospital Virgen de la Salud, Avda. Barber 30, 45004-Toledo, Spain; 2 Department of Pathology, Hospital Virgen de la Salud, Avda. Barber 30, 45004-Toledo, Spain; 3 Department of Pathology, Hospital Clinic, Barcelona, C/Villarroel, 170, 08036-Barcelona, Spain; 4 Department of Pathology, Complejo Hospitalario Xeral-Cies, C/Pizarro, 22, 36204-Vigo, Spain; 5 Banco de Tumores, Spanish National Cancer Centre (CNIO), c/Melchor Fernéndez Almagro 6, 28029-Madrid, Spain; 6 Neurosurgery, Hospital Virgen de la Salud, Avda. Barber 30, 45004-Toledo, Spain

## Abstract

**Background:**

Conventional cytogenetic and comparative genomic hybridization (CGH) studies in brain malignancies have shown that glioblastoma multiforme (GBM) is characterized by complex structural and numerical alterations. However, the limited resolution of these techniques has precluded the precise identification of detailed specific gene copy number alterations.

**Results:**

We performed a genome-wide survey of gene copy number changes in 20 primary GBMs by CGH on cDNA microarrays. A novel amplicon at 4p15, and previously uncharacterized amplicons at 13q32-34 and 1q32 were detected and are analyzed here. These amplicons contained amplified genes not previously reported. Other amplified regions containg well-known oncogenes in GBMs were also detected at 7p12 (*EGFR*), 7q21 (*CDK6*), 4q12 (*PDGFRA*), and 12q13-15 (*MDM2 *and *CDK4*). In order to identify the putative target genes of the amplifications, and to determine the changes in gene expression levels associated with copy number change events, we carried out parallel gene expression profiling analyses using the same cDNA microarrays. We detected overexpression of the novel amplified genes *SLA/LP *and *STIM2 *(4p15), and *TNFSF13B *and *COL4A2 *(13q32-34). Some of the candidate target genes of amplification (*EGFR, CDK6, MDM2*, *CDK4*, and *TNFSF13B*) were tested in an independent set of 111 primary GBMs by using FISH and immunohistological assays. The novel candidate 13q-amplification target *TNFSF13B *was amplified in 8% of the tumors, and showed protein expression in 20% of the GBMs.

**Conclusion:**

This high-resolution analysis allowed us to propose novel candidate target genes such as *STIM2 *at 4p15, and *TNFSF13B *or *COL4A2 *at 13q32-34 that could potentially contribute to the pathogenesis of these tumors and which would require futher investigations. We showed that overexpression of the amplified genes could be attributable to gene dosage and speculate that deregulation of those genes could be important in the development and progression of GBM. Our findings highlight the important influence in GBM of signaling pathways such as the PI3K/AKT, consistent with the invasive features of this tumor.

## Background

Glioblastoma multiforme (GBM) is the commonest and most malignant of the primary central nervous system tumors in the human adult. Mean survival of GBM patients treated with the current standard therapy is approximately one year [1].

Glioblastomas, like other solid tumors, are characterized by changes in the expression of oncogenes and tumor suppressor genes, often as a consequence of numerical chromosomal abnormalities (genomic amplifications, gains, and losses) that occur during the tumoral process. Conventional and molecular cytogenetic techniques, such as comparative genomic hybridization (CGH), have led to the identification of recurrent genomic copy number changes that play an important role in the malignancy of GBM. Aberrations that occur with high frequency include gains of chromosomes 7, 19, and 20, and losses of chromosomes 6q, 9p, 10, 13q, and 14q [2,3]. Nevertheless, the low resolution of these techniques, which is restricted to the chromosome level, together with the large number of genes located within these regions, makes difficult the identification of candidate genes.

High-level DNA copy number changes in tumors are restricted to chromosome regions that show more than 5- to 10-fold copy number increases (regions of amplification, or amplicons). Some of these amplicons contain well-known oncogenes that are also overexpressed. While this is the case for oncogenes associated with the development of GBM, such as *Epidermal Growth Factor Receptor *(*EGFR) *(7p12), *Cyclin-Dependent Kinase 4 *(*CDK4*) (12q14), and the human homolog of the *Mouse Double Minute 2 *(*MDM2) *(12q15) [2-4], other regions of amplification and/or other relevant genes located within these or other regions remain unknown or incompletely described.

New high-throughput genomic technologies, such as cDNA microarray CGH [5], use conventional cDNA microarrays that are normally used in expression profiling, to examine genomic copy number imbalances. In this way, thousands of genes can be reviewed in a high-resolution analysis to define amplicons and identify candidate genes showing recurrent genomic copy number changes. Parallel expression profiling experiments then allows the identification of relevant target genes whose aberrant expression could suggest its involvement in the pathogenesis of the tumors [6-10].

The objective of our study was to define at high resolution regions of amplification in GBMs, and through integration of copy number and gene expression data, to identify possible candidate target genes that could give insights into the pathology of GBM. In addition, we also aimed to analyze in detail the gene copy number changes associated with these tumors, since this is not feasible using classic chromosomal CGH.

For that purpose, we surveyed for changes in DNA copy number and expression levels throughout the genomes of 20 primary GBMs by using cDNA microarray CGH and expression profiling experiments. The most significant alterations found were validated in additional series of primary GBMs using locus-specific fluorescence in situ hybridization (FISH), and immunohistochemical analyses.

## Methods

### Patients and tissue samples

The genomic-profiling study involved 20 cases of primary GBMs. Expression profiling experiments were carried out in 17 of the GBMs for which RNA material was available. Thirteen of the patients were males and 7 were females with a mean age of 61 years (range, 39 to 81 years). The clinical information of the patients is summarized in Table 1.

**Table 1:** Clinical and molecular data of the patients.

**Case**	**Sex**	**Age**	**Site**	**OS**	**Status**	***7p EGFR***	***1q***	***4p/4q***	***7q***	***12q CDK4***	***12q MDM2***	***13q***	***-10q***
**C29**	M	39	T	46	A	**A**							
**C34**	F	81	P-O	4	A	**A**							**L**
**C35**	M	51	T	3	D	**A**							**L**
**C35b**	F	66	P	1	A	**A**	**A**						**L**
**C36**	F	55	T-P	18	A	**A**							**L**
**C38b**	F	45	T-P	2	A	**A**							**L**
**C42**	M	70	T-P-TA	2	D	**A**							
**C47**	M	60	T	10	A	**A**							
**C33**	F	61	T	16	D	**A**			**A**		**A**		**L**
**C26**	M	52	F-T-P	3	D	**A**				**A**			**L**
**C36b**	M	41	T	10	A					**A**	**A**		**L**
**C48**	M	74	T	7	D					**A**		**A**	
**C28**	F	77	T	4	D							**A**	
**C39b**	M	53	F	0	D			**A**				**A**	**L**
**C32**	M	60	T-P	8	D						**A**		
**C30**	M	79	T-P	1	D								
**C31**	M	79	O	6	D								
**C37b**	M	75	T	0	D								**L**
**C43**	F	53	P-O	3	A								
**C46**	M	59	T	9	D								

**Clinical data**: sex (M male, F female), tumor site (T temporal, P parietal, O occipital, F frontal and T thalamus), overall survival (OS) in months, and status (D death, A Alive).

**Molecular data**: (A) amplification, (L) loss.

To validate our results, 111 primary GBMs were arrayed in tissue microarrays (TMAs) and subjected to immunohistochemical and FISH analyses. Samples were collected from Virgen de la Salud Hospital (Toledo, Spain), Clinic Hospital (Barcelona, Spain) and Xeral-Cies Hospital (Vigo, Spain). All samples were reviewed by means of tissue sections stained with hematoxylin and eosin (H&E) to verify tumor viability and confirm the diagnosis according to the WHO guidelines by M.M., T.R., C.F., and F.-I.C.

### cDNA microarray CGH and expression profiling

The cDNA microarrays used in this study (Oncochip v2.0) were purchased from Centro Nacional de Investigaciones Oncológicas [11]. These microarrays contained 27,454 cDNA clones, including 9,900 known genes and uncharacterized ESTs related to tumorigenesis.

High molecular weight genomic DNA from tumors and normal human lymphocytes (used as reference DNA) were extracted following standard phenol/chloroform purification protocols. CGH experiments on cDNA microarrays were performed as described [5,10,12]. Briefly, 20 μg of genomic tumoral and reference DNAs were digested for 14–18 hours with *Alu*I and *Rsa*I (Life Technologies, Inc., Rockville, MD) and purified by phenol-chloroform extraction. Six μg of purified digested tumor DNA and reference DNA were labeled with Cy5-dUTP and Cy3-dUTP (Amersham Biosciences, Piscataway, NJ), respectively, using the Bioprime Labeling Kit (Life Technologies, Inc.). Labeled tumor and sex-matched reference DNA were co-hybridized at 58°C for 14–16 hours. Post-hybridization washes were carried out in 2×S SC/0.03% SDS at hybridization temperature, 1× SSC and 0.2× SSC at room temperature for 5 minutes each.

For expression profiling experiments total RNAs were extracted with Tri Reagent (Molecular Research Center, Cincinnati, OH) and amplified using a T7-based method, as previously described (11). Five μgr of total RNA were used to produce double-stranded cDNA (Superscript Choice System, Life technologies, Inc.) and amplification of mRNAs was performed using the Megascript T7 in vitro transcription kit (Ambion, Austin, TX). A pool of aRNAs obtained from the Universal Human RNA (Stratagene, La Jolla, CA) was used as a standard reference in all hybridizations. Test or reference amplified RNAs (aRNAs) were labeled with fluorescent Cy5 and Cy3, respectively, and hybridized at 42°C for 15 hours. Two control RNAs obtained from non-tumoral brain (one of them from Stratagene) were used for normalization purposes.

After hybridizations, slides were scanned using an Axon GenePix 4100A confocal scanner. Image analysis was performed using GenePix Pro 6.0 software (Axon Instruments Inc., Union City, CA). Cy5/Cy3 fluorescence ratios were normalized for each microarray using the print-tip loess method and background subtraction with the Diagnosis and Normalization Array Data (DNMAD) tool [13].

### Microarray data analysis

Data were preprocessed using Gene Expression Preprocessing Analysis Suite (GEPAS) [14] [see Additional file 1]. Cut-off points for defining gains and losses of genetic material in the test hybridizations were established as reported before [7,10]. The mean log_2_-transformed ratios derived from the self *versus *self experiments of normal genomic DNA in control hybridizations allowed us to establish the cut-off points for defining gains and losses. A value of the mean ratio +/- two standard deviations showed a normal range of variation corresponding to log_2_-transformed values of -0.42 to 0.42. In order to ensure a fluorescence ratio of gain or loss, we considered gene gain to be when log_2 _ratios were ≥ 0.5, and gene loss to be when log_2 _ratios were ≤-0.5. Log_2 _fluorescent ratios ≥ 2 were considered to represent gene amplification.

### Fluorescence in situ hybridization and immunohistochemistry on TMAs

TMAs were constructed using formalin-fixed paraffin-embedded archival tissue blocks as reported [15]. Five non-tumoral controls (4 normal brain and one tonsil tissue samples) were included. H&E-stained full sections from each donor block were used for morphological selections of the representative areas of each case.

FISH assays were performed as described previously [15] using gene-specific and control BAC clones selected from the EnsEMBL [16] and UCSC [17] databases (Table 2). Gene probes were labeled with SpectrumOrange-dUTP (red) and control probes with SpectrumGreen-dUTP (green) using the CGH Nick Translation Kit (Vysis). Hybridizations were done overnight at 37°C after deparaffinization of 4-μm-thick sections of the TMAs, target retrieval by pressure-cooking with 1 mM EDTA for 10 minutes, and pepsin digestion (4 mg/ml at 37°C for 30 minutes). After post-hybridization washes, tissue was counterstained with DAPI in antifade solution (Oncor, Gaithersburg, MD).

**Table 2:** Clones used for validation by FISH.

**Gene name**	**Gene-specific-BAC clones**	**Location**	**Control clones**	**Location**
*EGFR*	RP11-339F13	7p12	p275α7^a^	7 cen
*CDK4*	RP11-571M6	12q13-q14	RP11-467M14	12p13
*MDM2*	RP11-611O2, RP11-450G15	12q15	RP11-467M14	12p13
ESTs on 13q	RP11-94P17, RP11-406G20	13q32.2		
	RP11-364F8, RP11-151A6, RP11-113J24	13q33.3		

^a ^plasmid probe located at the centromere (cen) of chromosome 7. The specificity and location of the probes were confirmed by FISH on normal metaphases before hybridization on the TMAs. When clones were available, and in order to increase the signal, two or three BAC clones around the test gene or EST were hybridized together. Control FISH experiments on normal interphase nuclei were performed beforehand in order to ensure a unique signal when two BAC clones were hybridized together. BAC clones on 13q were purchased from the BACPAC Resources Center (Children's Hospital Oakland Research Institute, CHORI). All other clones were generously provided by Dr. Rocchi.

Fluorescence signals were scored (Y.R., B.M.) in accordance with previous reports [18]. In each sample, only well-defined nuclei were analyzed, and the numbers of single-copy gene and control probe signals were scored. Tumors were considered as amplified when five or more unbalanced gene copies, or more than three times as many gene signals as control signals were found in more than 5% of tumor cells.

Immunophenotypic analysis was performed on deparaffinized TMA sections. For antigen retrieval, a heating step in a solution of 10 mM sodium citrate buffer at pH:6 in a pressure cooker was included before incubation with antibodies. Tissues were immunohistochemically stained by the Labeled Streptavidin Biotin (LSAB) (DAKO, Glostrup, Denmark), or alkaline phosphatase-conjugated EnVision (DAKO) method, using the TechMate 500 (DAKO) automatic immunostaining device. The primary antibodies used were EGFR, MDM2 (Oncogene Research Products, Boston, MA) and TNFSF13B (BAFF or BlySS). Tumors were considered positive when membranous (EGFR), nuclear (MDM2), and membrane-bound or cytoplasmic (TNFSF13B) staining was observed in ≥ 5% of the tumor cells.

## Results

### Impact of copy number alteration on gene expression

**Figure 1:**
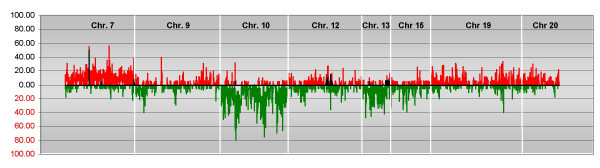
**Frequency of copy number change**. (**A**) Frequency of gene amplification (black), gain (red), and loss (green) in the 20 GBMs relative to their map position in the EnsEMBL database (calculated as the number of GBMs showing amplified, gained, or lost genes *vs*. the number of tumors analyzed for each gene). The white vertical bars represent the separation between chromosomes. Only the most representative chromosomes are shown. Map positions for each cDNA clone were obtained from the EnsEMBL database using the IDconverter tool [45].

The gene frequencies of gain and loss of genetic material found in the 20 primary GBMs were calculated, and plotted relative to the position along the chromosome (Figure 1). Chromosomes 7, 19, and 20 most frequently showed gains in copy number, while chromosomes 10 and 13 most frequently had losses (Table 3).

**Table 3:** Frequent copy number and expression altered genes^1^.

**GAINED AND OVEREXPRESSED GENES**				
**SYMBOL**	**GENE DESCRIPTION**	**%**	**MEDIAN**	**LOCATION**

*ATP2B4*	*ATPase, Ca++ transporting, plasma membrane 4*	20.00	1.17	1q25-q32
*LOC153222*	*Adult retina protein*	25.00	1.32	5
*GBAS*	*Glioblastoma amplified sequence*	20.00	1.61	7p12
*GUSB*	*Glucuronidase, beta*	20.00	1.42	7q21.11
*RFC2*	*Replication factor C (activator 1) 2, 40kDa*	26.32	1.06	7q11.23
*MCM7*	*MCM7 minichromosome maintenance deficient 7 (S. cerevisiae)*	35.29	1.02	7q21.3-q22.1
*GIMAP6*	*GTPase, IMAP family member 6*	20.00	1.29	
*LR8*	*LR8 protein*	21.05	2.34	7q36.1
*APOE*	*Apolipoprotein E*	22.22	1.48	19q13.2
*EMP3*	*Epithelial membrane protein 3*	33.33	1.36	19q13.3
*FPR1*	*Formyl peptide receptor 1*	25.00	1.94	19q13.4

**LOST AND UNDEREXPRESSED GENES**				

**SYMBOL**	**GENE DESCRIPTION**	**%**	**MEDIAN**	**LOCATION**

*CYP1B1*	*Cytochrome P450, family 1, subfamily B, polypeptide 1*	21.05	-2.87	2p21
*TDE2*	*Tumor differentially expressed 2*	23.53	-1.86	6q22.32
*OPTN*	*Optineurin*	41.18	-1.86	10p14
*DNAJC12*	*DnaJ (Hsp40) homolog, subfamily C, member 12*	25.00	-1.16	10
*SIRT1*	*Sirtuin (silent mating type information regulation 2 homolog) 1*	27.27	-1.27	10q22.1
*PPP3CB*	*Protein phosphatase 3 (formerly 2B), catalytic subunit, beta isoform*	35.00	-2.08	10q21-q22
*GHITM*	*Growth hormone inducible transmembrane protein*	37.50	-1.55	10q23.2
*CYP26A1*	*Cytochrome P450, family 26, subfamily A, polypeptide 1*	21.05	-1.08	10q23-q24
*LGI1*	*Leucine-rich, glioma inactivated 1*	45.00	-1.32	10q24
*SLK*	*STE20-like kinase (yeast)*	26.32	-1.11	10q25.1
*ADD3*	*Adducin 3 (gamma)*	68.42	-1.04	10q24.2-q24.3
*MXI1*	*MAX interactor 1*	40.00	-1.11	10q24-q25
*ATRNL1*	*Attractin-like 1*	30.77	-1.65	10
*KIAA1598*	*KIAA1598*	21.05	-1.83	10q26.12
*NDFIP2*	*Nedd4 family interacting protein 2*	33.33	-1.22	13q22.2
*MAP2K4*	*Mitogen-activated protein kinase kinase 4*	23.53	-1.30	17p11.2
*DSG2*	*Desmoglein 2*	31.25	-1.94	18q12.1
*TPTE*	*Transmembrane phosphatase with tensin homology*	22.22	-1.67	21p11

^1^Percentage of tumors showing copy number and expression alterations **(%)**. Median of expression log_2 _ratios of copy number altered genes **(median)**.

**Figure 2:**
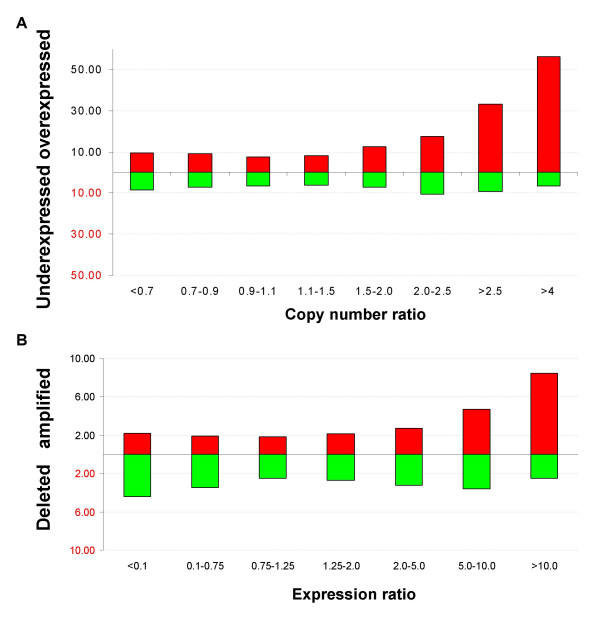
**Impact of gene copy number on global gene expression levels**. (**A**) Percentage of over- or underexpressed genes (*Y axis*) according to copy number ratios (*X axis*). (**B**) Percentage of amplified and deleted genes according to expression ratios.

The global effect of copy number alterations on gene expression was evaluated in 17 of the primary tumors. Up to 33% of gained genes (>2.5-fold change in copy number), or up to 56% with >4-fold, were overexpressed. Nevertheless, 8% of the genes with normal copy numbers were overexpressed (Figure 2A). Conversely, approximately 8% of the transcripts with high-level expression (>10-fold) showed amplification (Figure 2B).

### Gene amplifications in primary GBMs

Amplicons were identified on chromosomes 1, 4, 7, 12, and 13 due to the presence of more than four contiguous amplified genes. *EGFR *(7p12) was the most commonly amplified gene (7p12), showing amplification by microarray CGH in 50% (10/20) of the GBMs (Table 1).

**Figure 3:**
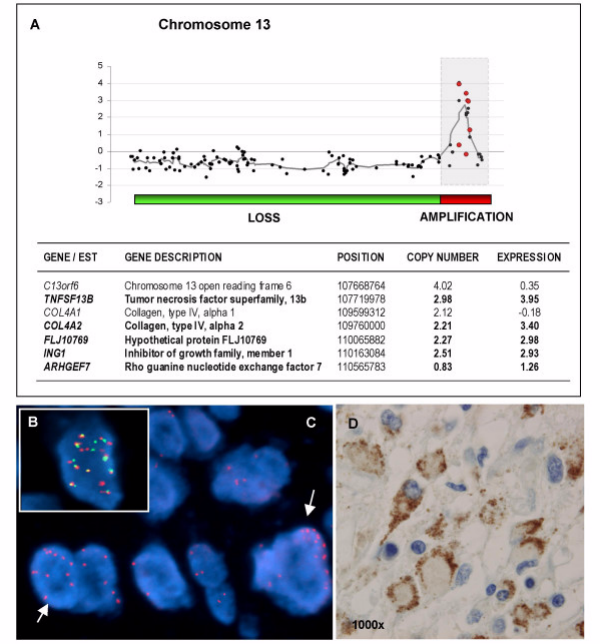
**Amplification at 13q**. (**A**) DNA copy number and expression log_2 _ratios (black and red dots, respectively) of tumor 39b plotted according to the map position. Moving average of log_2_-genomic ratios over five neighboring genes are plotted and shown with a grey line. In the table below, amplified and overexpressed genes (in bold), with their corresponding log_2_-ratios, are detailed. Lost and amplified regions are indicated by green and red bars, respectively, under the graph. The amplified region at 13q32-34 is indicated by a grey square. (**B**) Amplification at 13q observed in one of the tumors of the TMA by using BAC probes located at 13q32.3 (labeled in red) and 13q33.2 (labeled in green). (**C**) Amplification on 13q32.3 in another GBM (BAC probes labeled in red). Amplified cells are indicated by arrows. (**D**) Photomicrograph of tumour tissue with positive expression for TNFSF13B showing cytoplasmic pattern (original magnification ×1000).

The amplicon detected on chromosome 13 in tumor 39 (Figure 3A) contained contiguous amplified clones covering a region of about 2.5 Mb at 13q32-34 that included *TNFSF13B *(a ligand of the tumor necrosis factor superfamily), and collagen type IV genes (*COL4A1 *and *COL4A2*). Among the amplified genes or ESTs, the most important fold-changes in expression levels were those of *TNFSF13B*, *COL4A2 *and *FLJ10769*. In addition, two other tumors had centromeric-amplified clones including ESTs AA706834, AA994053, and AI093016. However, these ESTs were not overexpressed.

To determine the frequency of 13q amplification in a larger series of tumors, we carried out FISH assays in an independent set of 111 GBM samples. We found 8,5% of tumors (6/70) showing amplification (Figure 3B, 3C). In this same series, we examined the protein expression of *TNFSF13B *as one of the putative target genes of the 13q amplification. We detected TNFSF13B immunostaining positivity in 20,6% of the samples (20/97). Half of the 13q-amplified tumors (3/6) showed TNFSF13B positive expression, and 7 out of 59 (11,8%) non 13q-amplified tumors showed TNFSF13B positivity (Figure 3D).

**Figure 4:**
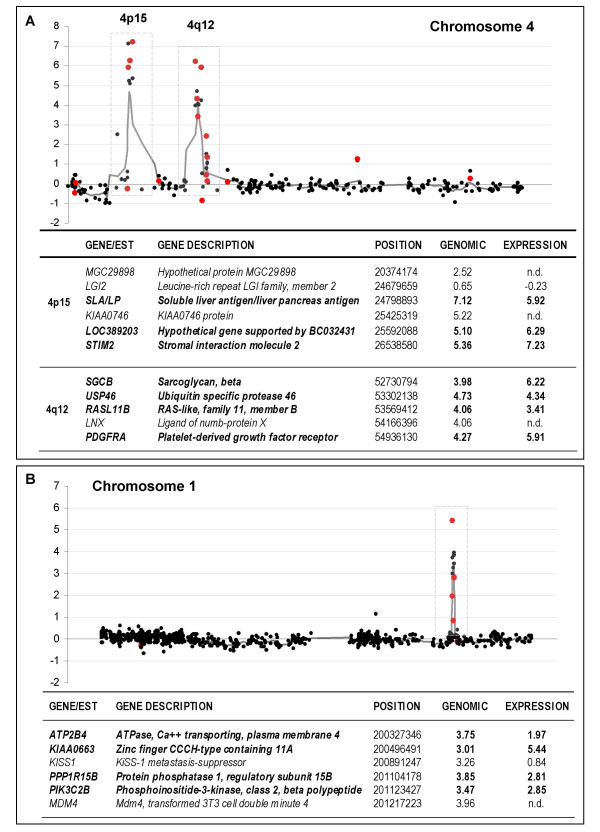
**Amplification at chromosomes 4 and 1**. (**A**) DNA copy number ratios and expression log_2 _ratios (black and red dots, respectively) plotted with respect to their map position obtained for chromosome 4 of tumor 39b, and (**B**) for chromosome 1 of tumor 35b. Average log_2 _genomic values over five neighboring genes are shown with a grey line as a function of the location of the clones. Described in the corresponding tables below each graph are the amplified and overexpressed (in bold) genes together with their corresponding copy number and expression log2 values.

Amplicons were also detected on 1q, 4p, 4q, 7q, and 12q. Chromosome 4 had two separated regions of amplification (Figure 4A), one of about 6 Mb at 4p15, and the other of approximately 2.2 Mb at 4q12. The 4p15 amplicon is described here for the first time and contained several genes and ESTs not previously reported as being amplified in GBMs. The amplicon at 4q12 contained *PDGFRA*, whose amplification in GBMs is already well known. Among the amplified genes, *SLA/LP*, *LOC389203*, *STIM2*, *SGCB*, *RASL11B*, *and PDGFRA *seem to respond to gene dosage, presenting high fold-changes in expression levels (Figure 4A). Likewise, amplification of several contiguous clones on chromosome 1q32 included *ATP2B4*, *KIAA0663*, *KISS1*, *PPP1R15B*, *PIK3C2B*, and *MDM4*. Among them, *ATP2B4, KIAA0663, PPP1R15B, PIK3C2B *were overexpressed in the expression analyses (Figure 4B).

Chromosome 12 showed two regions of amplification at 12q13-15 (Table 4), one of about 180 Kb at 12q13 containing *CDK4 *(10%, 2/19) and another one of about 700 Kb containing *MDM2 *(15%, 3/20) at 12q14.3-q15. These results were validated by FISH analyses with specific probes containing *CDK4 *or *MDM2 *and immunohistochemical analyses for MDM2 onto paraffin sections of the samples in a set of 111 primary GBMs, allowing confirmation of the microarray results (Table 1). We found alterations (either amplification or overexpression) of MDM2 and CDK4 in 11% of the tumors for which data were available (11/98 and 7/66, respectively). Except for one tumor, the two regions were not amplified simultaneously. These results suggest that *MDM2 *and *CDK4 *may be independently amplified in most GBM tumors and confirm those of other authors [19]. Finally, amplification at 7q included *PEX1*, and *CDK6 *(7q21) as overexpressed putative amplification targets (Table 5).

**Table 4:** Amplicon at 12q.

	**SYMBOL**	**GENE NAME**	**POSITION**	MEDIAN* GENOMIC	MEDIAN* EXPRESSION
	
**AMPLICON 1**	*SLC26A10*	*Solute carrier family 26, member 10*	56290368	0.49	0.44
	*GALGT*	*UDP-N-acetyl-alpha-D-galactosamine*	56305945	1.73	0.33
	***SAS***	***Sarcoma amplified sequence***	56425051	**0.49**	**2.92**
	***CDK4***	***Cyclin-dependent kinase 4***	56428272	**2.44**	**4.12**
	***CYP27B1***	***Cytochrome P450, family 27, subfamily B, polypeptide 1***	56442389	**2.35**	**1.93**
	*TSFM*	*Ts translation elongation factor, mitochondrial*	56462849	1.02	-0.26
	*AVIL*	*Advillin*	56477704	1.84	0.53
	
**AMPLICON 2**	*GNS*	*Glucosamine (N-acetyl)-6-sulfatase*	63393491	0.48	0.47
	***CGI-119***	***CGI-119 protein***	64817459	**0.94**	**5.38**
	*IFNG*	*Interferon, gamma*	66834816	0.76	-0.02
	***MDM2***	***Mdm2, transformed 3T3 cell double minute 2***	67488247	**2.72**	**1.64**

* Median of genomic or expression log_2 _ratios of gained genes.

**Table 5:** Amplicon at 7q.

**SYMBOL**	**GENE DESCRIPTION**	**POSITION**	**GENOMIC**	**EXPRESSION**
*PFTK1*	PFTAIRE protein kinase 1	89870462	1.68	-0.58
***FZD1***	**Frizzled homolog 1 (Drosophila)**	**90538434**	**3.05**	**2.91**
***PEX1***	**Peroxisome biogenesis factor 1**	**91760991**	**3.13**	**4.04**
*CDK6*	Cyclin-dependent kinase 6	91878888	1.53	0.43

Genomic and expression log_2 _ratios of gained genes.

## Discussion

The overall impact of copy number on gene expression analyzed in GBMs reflects the importance of recurrent gene copy number changes in the development and progression of these brain tumors. Our results in GBMs extend previous studies in breast and prostate cancers [10,20,21], and confirm that the effects of gene copy number on expression levels were more relevant for high-level amplifications on a gene-by-gene analysis (56% of highly-gained genes were overexpressed).

Gene amplification is regarded to reflect genetic instabilities in solid tumor cells [22]. It has been proposed that activation of proto-oncogenes by amplification plays an important role in the development of many human solid tumors. Therefore, detection of specific gene amplifications in tumor cells can lead to the identification of genes putatively involved in growth control and tumorigenesis.

While only a few candidate genes could be investigated at a time in previous studies [4,23,24] we have used cDNA microarray technology to search the whole genome for gene copy number alterations in GBMs. Our results from the approximately 10,000 genes and ESTs related to the tumoral process that could be analyzed in this study, confirm the previous conclusion that *EGFR *is the principal oncogene amplified in GBMs. However, in this study several other chromosomal regions showing gene amplification have also been detected in GBMs at 1q, 4p, 4q, 7q, 12q, and 13q. Amplification at 13q32-34 has been reported before from chromosomal CGH studies in malignant gliomas, cell lung carcinoma, head and neck squamous carcinoma, and systemic lymphomas [20,25-29]. Here we have used microarray CGH to study this region at higher resolution (gene level) and have narrowed down the region to 2.5 Mb, showing that this amplification could affect a small fraction of GBMs (8%). Several known genes and uncharacterized ESTs are contained in the 13q amplicon. Among these, *COL4A2 *has also been found to be overexpressed in our study and in other microarray expression-profiling studies of GBM biopsies and GBM cell lines [30-32]. We speculate that this overexpression may have been caused by amplification of *COL4A2*, which may be a putative target of the amplicon. In agreement with this hypothesis, very recently, Tso and coworkers [33] have shown that *COL4A2 *is one of 15 highly expressed genes that is shared between primary and secondary GBMs. In addition, this gene was found to be involved in glioma progression and associated with vascular proliferation. However, immunohistochemical analyses revealed that collagen IV is mainly expressed in association with the tumor blood vessels and not from the tumor cells [32], suggesting that the *COL4A2 *overexpression detected in GBMs may not be as a result of gene amplification in tumoral cells. This may point out to *TNFSF13B *as a likely candidate for the target of amplification. Immunohistochemical analyses of 111 primary GBMs revealed that TNFSF13B could be affected in about 20% of the tumors. The TNFSF13B ligand (or BAFF) is a member of the TNF cytokine family that activates nuclear factor (NF)-κB, phosphatidylinositol-3 kinase (PI3K)/AKT, and mitogen-activated protein kinase (MAPK) pathways in myeloma multiple cells, and induce strong up-regulation of Mcl-1 and Bcl-2 antiapoptotic proteins [34].

On another hand, the gene *IRS2 *(which codes a cytoplasmic adaptor protein that facilitates intracellular signal transduction) is also located within the amplified region close to *COL4A2 *and *TNFSF13B*. Although we have no data available for this gene, one study suggests that *IRS2 *is a novel but rare amplification target at 13q34 in GBMs [35]. Thus, we propose here that genes such as *COL4A2 or TNFSF13B *could be additional putative targets for the 13q amplicon, and therefore would warrant further detailed analyses in GBMs.

It is of particular note that two of the tumors showing this amplification in the telomeric region of chromosome 13 had loss of the rest of the chromosome (Figure 3A), as was also described by Weber and coworkers in the cases in which amplification was revealed by chromosomal CGH [36]. This finding is consistent with the finding that gene amplification may be accompanied by loss of genetic material in the proximity of the amplification site [37].

Chromosome 4 has two amplicons, located close together. One contains *PDGFRA *as the putative target of the amplicon, and the other contains *SLA/LP*, *STIM2*, and two ESTs. To our knowledge, this is the first report of amplification of *SLA/LP *and *STIM2 *genes, which are both overexpressed. However, further analyses are required to identify the amplification gene target(s) and to determine the relevance of this novel amplification in GBM. *STIM2 *codes for a transmembrane phosphoprotein whose structure is unrelated to that of any other known protein [38] and whose biological function has not been thoroughly studied. STIM1 is the other member of this family of proteins and it is thought to regulate cell growth control and function within a signaling cascade, although the precise pathway is not known [38].

There is controversy concerning the amplification on chromosome 1, as to whether 1q32 has two independent amplification targets or a single one affecting both *MDM4 *and *CNTN2 *genes [39-41]. We observed amplification covering a small region of about 800 Kb that excluded *CNTN2*. Thus, our results support the proposal of Riemenschneider and coworkers that *MDM4 *is the main amplification target gene at 1q32 [40]. However, other genes among those contained in the amplicon, such as *PIK3C2B*, which encodes a catalytic subunit of the PI3K, could be of importance in GBMs. This gene was also found amplified and overexpressed in GBMs by others [32,41] and has a crucial role in the PI3K/AKT signaling pathway, which regulates a number of cellular processes such as cell growth and proliferation, apoptosis, migration and invasion, and angiogenesis [39]. Thus, our study excludes *CNTN2 *and shows that other interesting candidate genes together with *MDM4 *may be important gene targets for this 1q32 amplification.

Chromosome 7 was the most frequently gained chromosome in GBMs, as already known. Our results showed a large number of overexpressed gained-chromosome 7 genes which may suggest the importance of the complete gain of this chromosome in primary GBMs. Further studies, however, should be carried out to assess the importance of chromosome 7-candidate genes, other than *EGFR*, in the pathogenesis of GBM. Likewise, chromosome 10 was the most frequently lost chromosome in GBMs. The most frequently lost and underexpressed genes mapped at 10q and involved candidate genes in gliomas such as *ADD3*, between others. Downregulation of *ADD3 *expression was associated with increased migratory activity of human glioma cells *in vitro *[42], and decreased expression of *ADD3 *has been described in astrocytomas [43]. Chromosome 19 also showed frequent gene gains, mainly located on 19q. One of the possible candidates located in this chromosome could be *FPR1 *due to this gene is expressed in malignant glioma and appears to mediate motility, growth, and angiogenesis of GBM [44].

## Conclusion

In summary, our results show that the cDNA microarray CGH technique in parallel with expression profiling allows the comprehensive, rapid and reliable analysis of the whole genome in GBM tumors and enables the refined and detailed study of amplicons and regions of recurrent copy number change. This approach makes it possible to identify putative glioma oncogenes/tumor-suppressor genes that may deserve further investigation. Our findings highlight the important influence in GBM of signaling pathways such as the PI3K/AKT, consistent with the invasive features of this tumor. In this context, we identify candidate target genes of amplification that may help to direct therapeutics for the treatment of GBM.

## Authors' contributions

YR carried out selection of the GBM samples, performed nucleic acid extraction, DNA and RNA hybridizations onto the microarrays, FISH experiments, and participated in the discussion of the results. MM, TR, CF, and FIC carried out histopathological analyses and pathological diagnosis. MM and FIC participated in the design of the study, and in the analysis and discussion of the results. EG participated in selection of the samples, DNA and RNA extraction, and carried out construction of the tissue arrays. AR-L and J-LH-M recovered clinical data of the patients and carried out clinical diagnostics. PM participated in the design and discussion of the results BM carried out statistical analysis of the microarray results, conceived of the study, drafted the manuscript, participated in its design, and carried out coordination. All authors read and approved the final manuscript.

## Supplementary Material

Additional file 1 Genomic and expression row data. Genomic and expression row data of the GBM tumors. Gene symbol, gene description and localization are provided. Click here for file
